# Find the Gap: AI, Responsible Agency and Vulnerability

**DOI:** 10.1007/s11023-024-09674-0

**Published:** 2024-06-05

**Authors:** Shannon Vallor, Tillmann Vierkant

**Affiliations:** 1 School of Philosophy, Psychology and Language Sciences University of Edinburgh, Edinburgh, Scotland; 2 Edinburgh Futures Institute, University of Edinburgh, Edinburgh, Scotland

**Keywords:** Reactive attitudes, Agency cultivation, Moral responsibility, Autonomous systems, Problem of many hands, Vulnerability gap

## Abstract

The *responsibility gap*, commonly described as a core challenge for the effective governance of, and trust in, AI and autonomous systems (AI/AS), is traditionally associated with a failure of the epistemic and/or the control condition of moral responsibility: the ability to know what we are doing and exercise competent control over this doing. Yet these two conditions are a red herring when it comes to understanding the responsibility challenges presented by AI/AS, since evidence from the cognitive sciences shows that individual humans face very similar responsibility challenges with regard to these two conditions. While the problems of epistemic opacity and attenuated behaviour control are not unique to AI/AS technologies (though they can be exacerbated by them), we show that we can learn important lessons for AI/AS development and governance from how philosophers have recently revised the traditional concept of moral responsibility in response to these challenges to responsible human agency from the cognitive sciences. The resulting instrumentalist views of responsibility, which emphasize the forward-looking and flexible role of agency cultivation, hold considerable promise for integrating AI/AS into a healthy moral ecology. We note that there nevertheless *is* a gap in AI/AS responsibility that has yet to be extensively studied and addressed, one grounded in a relational asymmetry of *vulnerability* between human agents and sociotechnical systems like AI/AS. In the conclusion of this paper we note that attention to this vulnerability gap must inform and enable future attempts to construct trustworthy AI/AS systems and preserve the conditions for responsible human agency.

The *responsibility gap* has long been viewed as a core challenge for the effective governance of autonomous systems (hereafter, AS). The concept of an autonomous system can be defined in many ways, but for the purposes of this paper we generally refer to “a system involving software applications, machines, and people, that is able to take actions with little or no human supervision.”^1^ While the literature is split on the nature and true extent of responsibility gaps, they are typically attributed to a failure of the epistemic and/or the control condition of moral responsibility.^2^ That is, they are thought to arise when no moral agent in the right relation to an AS action has the ability to know what the system is doing, and/or exercise competent control over this doing.

It is widely agreed that AS today cannot be moral agents, that is, they cannot be morally responsible for their own actions. Yet we argue that this is not because AS are mere machines. Certainly an autonomous machine assemblage of any sort that exists today, encompassing the hardware, data, trained mathematical model and other system software, falls short of the criteria for responsible moral agency. At a minimum, these include the capacities for moral awareness, reflection, understanding, motivation, deliberation and judgment. However, taking a *sociotechnical systems approach* to AS (Johnson 2005) makes clear that the boundary around such systems includes not only hardware and software components, but also many human developers, deployers, users and regulators upon whom the system’s capabilities and operations depend. ‘Autonomy’ in a machine refers to the degree of a system’s ability to operate without a human ‘in the loop’ of a given task, but humans do not actually move all the way out of the functional system; they only move ‘out of the loop.’ Even today’s most advanced AS, powered by large, adaptive and highly complex machine learning models, typically require extensive human support to sustain system functioning.^3^

Yet responsibility gaps still arise in AS; not because humans are out of the picture but, as we argue, because the human agents within the system increasingly do not stand in the right kind of relation of moral vulnerability to those affected by the system (referred to in the literature and herein as *patients of responsibility*). This paper offers a novel philosophical diagnosis of the responsibility gap challenge for developing and governing trustworthy autonomous systems, rooted not in human failures to meet epistemic and control conditions (though these do compound AS responsibility challenges), but more fundamentally, in structural failures to properly embed reciprocal moral relations of mutual *vulnerability* between agents and patients within the responsibility practices that govern AS.

After outlining the nature of responsibility gaps, we argue in Section Two of the paper that the epistemic and control conditions are red herrings when it comes to understanding the responsibility challenges *uniquely* presented by AS, since evidence from the cognitive sciences shows that individual human actions present very similar responsibility challenges with regard to these two conditions. We then show in Section Three that recent instrumentalist accounts of moral responsibility succeed in addressing these challenges when they arise in individual human actions, by emphasizing the forward-looking and flexible role of responsibility practices in moral agency cultivation. We note that this solution also holds considerable promise for integrating AS into a healthy moral ecology.

However, in Section Four of the paper we observe a problem for this strategy, rooted in a concern not previously centered in the responsibility gap literature: the *vulnerability* gap between human beings and sociotechnical systems, including AS. The dynamics of mutual vulnerability between moral agents and patients play a driving role in the instrumental efficacy of responsibility practices. This is a problem for responsible and trustworthy AS because AS typically have a structural *in*vulnerability to human responsibility practices.

Moral invulnerability is intrinsic to the software and hardware components of an AS, which lack the moral and emotional experiences needed to be vulnerable to responsibility practices. Yet the bigger problem is that even the humans within an AS, who otherwise could bear the moral responsibility that a machine cannot, are often themselves cut off from the vulnerability needed to do so. The ‘many hands’ problem and the agency diffusion created by modern organizational structures of AS means that even the human actors in an AS rarely occupy the right roles to make them susceptible and answerable to the vulnerabilities of those patients of responsibility who may be harmed by AS actions.

In the conclusion of this paper we note that attention to this vulnerability gap must inform and enable future attempts to construct trustworthy autonomous systems and preserve the conditions for responsible human agency.

## The Responsibility Gap in the AI Literature

Matthias (2004) was the first to explicitly confront the challenge of so-called ‘responsibility gaps’ arising from what he then called ‘learning automata.’ Matthias was referring to an emerging class of machine learning systems, now commonplace, whose neural network architectures enable two features:

- Their actions or tasks can be executed with little or no need for proximate, real-time human supervision or direction (the *autonomy* component);
- Their performance responds and adapts to features of the task environment in ways that are not fully predetermined or predictable (the *learning* component).

Earlier forms of machine automation, such as the mechanical loom and 20th century manufacturing robots, embody only the first component. In such cases, assigning human responsibility for the machine’s actions and their consequences is mostly straightforward. If the system’s performance is predictable under expected task conditions, then responsibility for an autonomous system action that causes grave harm under those conditions (barring some unforeseeable mechanical breakdown) falls to those humans who culpably either failed to predict the harm, or failed to prevent it.

The problem that Matthias foresaw with adaptive machine learning technologies is that they would impede the ability of their designers or operators to fully understand or predict individual behaviours of a learning system, even under normal operating conditions, and even when the system’s aggregate performance is optimal (2004, 182). That is, AS unpredictability becomes the unavoidable price for superior performance overall. This can be due to the ‘intrinsic opacity’ of large deep learning models, the stochastic character of their outputs, their complex interactions with the environment and other systems, or some combination of these (Kroll 2017).

Matthias argues that it would be wrong to hold the human operator or manufacturer *morally* responsible for a harm that they could not possibly have foreseen or prevented. The machine assemblage itself is an unconscious mechanical artefact; it fails to meet even the most basic conditions of morally responsible agency.^4^ And yet to hold *no one* responsible for a harmful act of a system operating as designed seems to violate “our sense of justice and the moral framework of society” in ways that endanger social trust (2004, 177). This is the responsibility gap, which requires concrete design and governance interventions to address.

Matthias’ diagnosis has been controversial, but remains widely influential in academic, policy and design circles. The most obvious reply is to say that those deploying the technology are plainly responsible for the harm, because they deployed a system that is predictably unpredictable (Himmelreich, 2019; Johnson, 2011). Even if no human could have anticipated or prevented *that* specific harmful occurrence, someone had the ability to *not use an unpredictable machine for the task in the first place*. The response to this is normally to point out that as the social utility of such systems grows, along with improvements to their aggregate safety profile, such a policy of refusal is throwing the baby out with the bath water (Tigard, 2021a). On this view, what we need is a way to *bridge* the responsibility gap, so that AS can operate without undermining social trust and effective governance.

The law can only bridge part of the gap. As Heinrichs (2022) and others have pointed out, legal liability and moral responsibility are different social instruments. For some kinds of grave harms (imagine an autonomous vehicle that mows through a densely populated homeless camp to avoid a traffic jam) generic group liability such as a corporate fine, without targeted moral blame, will not satisfy victims or society’s sense of justice, or restore social trust. Where does that leave us? One family of suggestions involves enhancing the ability of humans to be more fully *answerable* to other humans for what AS do, in ways that restore the relational dynamics of responsibility (Coeckelbergh, 2020). Another approach taken by Kiener (2022) suggests that humans can retrospectively assign themselves as morally responsible agents, even when they are not conventionally blameworthy for or implicated by an AS action; but neither fully addresses why it matters *upon whom* responsibility falls; a point we return to in Section Three.^5^

Yet another suggestion by Nyholm (2018) is to redefine the conditions of agency and responsibility such that it becomes coherent to speak of ‘collaborative agency’ between human and machine systems. But as Heinrichs (2022) and others point out, in the end the burdens of responsibility in Nyholm’s account still fall on particular humans who may be unsatisfactory targets, if their causal contributions to a harm are smeared out so widely and thinly that punishing any individuals for their miniscule or obscure role appears disproportionate and unjust.^6^ At the other pole, holding ‘society’ at large responsible (Rahwan, 2018, Hellstrom 2013) gives us collective responsibility with no clear accountabilities or obligations, and will be of little solace to victims (Danaher, 2016).

Daniel Tigard (2021b) notes, with Kohler et al. (2017), that we already have ways of holding people morally accountable for unpredictable actions of non-responsible agents (such as bitey dogs). Forward-looking accountability measures can also be applied to AS by demanding that they *do better* in the future (2021b, 604). Tigard’s point is that “responsibility is a dynamic enterprise wherein we interact with an immense range of individuals,” who can and must be held responsible in different ways (604).

Our own view aligns with Tigard’s account in two key respects:

1. there is far more to moral responsibility than the knowledge and control of our actions upon which Matthias (2004) is focussed. As we make clear in Section Two, if a compromise of these two conditions were all it took to create a responsibility *vacuum*, we’d be unable to make any sense of moral responsibility in the human context, where knowledge and control gaps routinely arise. If there is a distinctive moral responsibility challenge from AS, it is not the knowledge or control problem – even if these are exacerbated by AS.
2. A further point of agreement is with Tigard’s view (2021a) that we can and should consider creative ways to make AS and other sociotechnical systems more answerable to those impacted by them, even as Tigard acknowledges that machines cannot answer for themselves *as moral agents*.

However, there are two key differences between ours and Tigard’s view:

1. Tigard is right that we routinely do make sense of moral responsibility in the human context, despite pervasive knowledge and control gaps, because responsibility is not a rigid designator of a natural phenomenon, but a flexible body of constructed social practices. However, Tigard does not offer a clear account of how these social practices can be justified. We turn in Section Three to the work of Manuel Vargas and Victoria McGeer, who show that these practices can be justified proleptically because they serve to hone responsible agents. We suggest that this approach can be adapted to a moral ecology that includes new types of agents and social relations, among them AS. Novel responsibility practices for AS actions can be justified, but *only* if they succeed over time in cultivating more responsible agency within the moral ecology that includes AS.
2. In Section Four we identify a serious obstacle to this end, which suggests that Tigard may be overly sanguine about our ability to work through responsibility gaps for AS. We note that creatively bridging responsibility gaps in human cases involves a dialogical process of agency cultivation through responsibility exchanges between *mutually vulnerable* parties. We argue that participation in such exchanges by AS is impeded not by our lack of knowledge or control of AS actions, but by a more significant and less noticed asymmetry of vulnerability in human-AS interactions. Thus, any attempt to bring AS into the responsibility circle by making AS answerable to people must first grapple with this vulnerability gap.

## Responsibility Gaps in the Case of Human Individuals

Before examining the AS vulnerability gap, however, we must consider how our theories of moral responsibility have already had to be revised in light of the knowledge and control gaps that trouble ideal notions of responsible human agency. For while responsibility gaps involving knowledge and control are not unique to AS, that does not mean that they are insignificant.

It is true that, as Tigard writes,

fully functional adult human beings also fail to provide adequate reasons for their decisions, say, due to implicit biases (Doris, 2015; Vargas, 2017) and this has not stopped us from demanding answers from them. (Tigard, 2021b, p. 602)

What Tigard fails to mention is that scholars sharing the view of Doris and Vargas (see Waggoner et al., 2022 for an overview) see a *problem* in the fact that we still demand answers from people, despite the fact that they are not able to give good reasons for their decisions. What we learn from the cognitive sciences about limited human control of, and epistemic access to, our decisions and actions *does* lead to serious worries about traditional accounts of moral responsibility. Some argue that these findings should make us reconsider whether humans can be responsible agents at all (King & Carruthers, 2012; Strawson, 2010). Others think that the findings require theorists of responsibility to come up with new and at least partially revisionary ideas in order to make our practices of holding each other responsible justifiable in the light of what we now know about the mechanisms of human agency.

An account of how to bridge responsibility gaps in AS would therefore do well to first take into account the revisions of the responsibility concept in the human case that have already been proposed as a reaction to the cognitive science challenge to the epistemic and control conditions. Let’s first revisit the empirical findings that have challenged the traditional responsibility concept.

There are a variety of experiments that put severe pressure on our naïve understanding of the conscious rational agent who is responsible by virtue of their conscious awareness and control. A large body of work suggests that our behaviour is far less dependent on the guidance of either stable character traits or conscious reasoning than traditional responsibility theories assume. The literature brought to fame by authors like Tversky and Kahneman (1974) revealed the unexpected role of implicit stereotype and appearance biases on our decisions, as well as the power of cognitive heuristics like recency or frequency of exposure to shape our beliefs and choices. Additionally, situational effects such as environmental cues and physiological states like hunger or fatigue seem to have an outsized effect on the choices we make, even when we are not consciously aware of these influences. While noting that the reproducibility and real-world impact of such findings remains a hotly contested issue, Mudrik et al. (2022) list a striking array of subliminal priming effects on decision making, just a small selection from the burgeoning literature:

…national flags led participants to vote for more central parties (Hassin et al. 2007), pro-social word primes increased donations by socially oriented participants (Andersson et al., 2017), disgusting faces reduced moral ratings of utilitarian decisions (Lim et al., 2017), and direct-gaze faces primes affected behavior in prisoner’s dilemma games (Luo et al., 2016) (Mudrik et al., 2022).

These findings from the cognitive sciences do not conclusively establish that the epistemic and control conditions for moral responsibility need to be abandoned or relaxed. As detailed by Waggoner (2022), defenders of these conditions can challenge the stability of the findings, or argue that the findings do not rule out our ability to satisfy the relevant epistemic and control conditions. Waggoner offers strong arguments why it is unlikely that the challenge from the empirical sciences can be ignored by responsibility theorists; on our view, responsibility *gap* theorists cannot ignore the challenge either.

For even if the ability to know and control what we are doing still plays a part in our capacity for being responsible, taken together these findings strongly suggest that this cannot be the whole story of how we become responsible, or of how our responsibility practices can be justified. The traditional picture of the responsible agent who knows why they are performing actions, is in full conscious control of those actions, and is appropriately responsive to reasons for acting, is a naïve one; it significantly underestimates the complexity and causes of real-life behaviour production. Vargas nicely sums up the overall problem with the lessons from the cognitive science literature:

The more global concern here is that the current state of psychological research on human agency suggests a much more contextually and socially embedded form of agency than is presupposed by going accounts of moral responsibility. (Vargas, 2017 225)

This evidence also suggests that we often don’t know the true reasons for which we have acted (Bortolotti 2018). This is a particular challenge for the narrow notion of responsibility as answerability that is favored by Tigard (2021a, 58), adopted from Shoemaker (2011), which requires the agent to produce their “reasons and justifications” for an act, or “an answer to the question of *why* an agent behaved in some way or other.^7^ The problem here is that the reasons that we do provide when asked may often be confabulated, or missing morally significant parts of the real causal story. Interestingly, we see similar phenomena in the latest class of AS built on generative AI language models, such as ChatGPT, which not only routinely confabulate falsehoods about the world, but often confabulate when challenged to explain their own mistakes and errors.

Nevertheless, as Tigard rightly points out, the apparent deficits in our abilities to fully satisfy the demands of responsible agency have not stopped us demanding answers from each other, or holding one another responsible. If these practices remain justified, how does the responsibility concept have to change in order to accommodate this new and still emerging picture of the human mind, and what does this change tell us about the path to bridging responsibility gaps with AS?

## Revising the Responsibility Concept: from Agency Possession to Agency Cultivation

Traditional atomist or ‘internalist’ accounts of responsibility often begin by working out what it would mean for an agent to be responsible. Armed with that clearly defined concept they then work out what it would take for agents to meet these requirements; that is, what qualities or capacities a responsible agent would *possess*. The problem with this approach is twofold; first, the lack of a stable philosophical consensus on precisely what it means to be responsible (Hurley, 2000; Vargas, 2021), and second, the aforementioned empirical challenges from the cognitive sciences to the knowledge and control conditions that have long been assumed to be central to any acceptable candidate for a consensus definition.

Philosophers who have recently diverged from the atomist or internalist approach instead develop versions of what has been termed the *agency cultivation* view (Vargas, 2021), influenced by a famous outlier in the traditional literature. Strawson (1962) argued that we should start our theorising about responsibility from the living practices of responsibility ascriptions and the reactive attitudes that come with them. There are two main features of this practice-based approach that the agency cultivation folk utilize to meet the cognitive science challenge. First, Strawson moved away from looking for a responsibility-enabling trait of the individual, focusing instead on the moral niche that agents operate in. Strawson develops a *relational* account of moral responsibility that begins with an investigation of the moral emotions such as indignation, gratitude and resentment that drive our social practices of holding each other responsible. On this view, at the heart of our responsibility practices is the simple fact that we typically cannot escape feeling, or being the target of, these emotions – the *reactive attitudes –*in relevant situations.

Agency cultivation theorists, like Strawson, view responsibility in relational and practice-based terms that take the reactive attitudes to be partly constitutive of the phenomenon of responsibility, rather than simply its effects. Furthermore, agency cultivation views deny that the ability to exercise responsible agency is a stable trait of individuals; instead, it consists in fragile intelligent capacities that require constant work. Being a responsible agent is more like being a competent mountain biker than like being lactose intolerant. The latter is a stable disposition, while the former is an actively maintained skill.

When these two features of the agency cultivation view are combined, they entail that some of our agency skills can only be acquired and maintained in a social context. As McGeer points out, many intelligent capacities cannot be acquired and sustained in isolation because they depend upon feedback from others:

The skills we need to develop are therefore skills in understanding and complying with a set of mutually shared and interpreted norms—often a matter of on-going negotiation and adjustment, particularly as the norms themselves are liable to change in response to changing social and environmental circumstances. Our capacities to engage in shared norm‐governed activities thus depends on our being tuned into how others respond to us, and we to them, thereby all doing our part to make and sustain the very norms that make such activities possible. (McGeer, 2019, p. 312)

These skill-based accounts naturally lend themselves to a different approach to the temporal dimension of responsibility. The emphasis here is forward looking and diachronic; cultivating a skill takes time, and the purpose of a responsibility practice is proleptic. It aims to form future responsible agents. Details vary quite considerably here between different accounts (see Waggoner et al., 2022), but they share the view that one of the main aims of responsibility practices is the cultivation of responsible agency.^8^ This stands in contrast to the backward-looking focus of traditional accounts, where the main purpose of a responsibility practice is to establish desert or blame for a specific event that happened in the past. While blame can also motivate and guide the delivery of retrospective justice, on the proleptic account, blame as a responsibility practice serves a forward-looking function. When blame is justified, appropriately directed, and effectively communicated, it brings about social and personal consequences that can make agents more aware of their character deficits, more apt in their moral reasoning and justifications, and more sensitive in the future to the moral stakes of exercising their agency without heeding others’ vulnerabilities.

We now are in a position to see how these agency cultivation approaches deal with the responsibility challenge from the cognitive sciences, which implies that humans have far less direct conscious access and direct control over the mechanisms that produce our behaviour than traditional accounts of responsibility require. On agency cultivation views, responsibility practices are less dependent on these features. Thanks to the social feedback loops driven by our moral emotions, responsible agency can be cultivated socially, even if the agent does not possess the necessary self-knowledge or control individually. On some agency cultivation views (McGeer & Pettit, 2015), even the seemingly backward-looking reactive attitudes of moral indignation and resentment associated with blame are best read proleptically, as functioning to motivate and inform the ongoing construction of future responsible agency.

As Vargas (2013) puts it, the purpose of responsibility practices is the progressive ‘building of better beings,’ that is, beings who *together* become more trustworthy and responsive to moral considerations over time. This can take place even if as individuals we lack some of the intrinsic, stable elements of self-knowledge and control traditionally thought central to responsible agency. As long as we are not *helpless to improve* the moral responsiveness of our agency, and we have the social feedback and relational supports needed to enable and sustain that improvement, then our practices of holding one another responsible remain wholly justified and coherent. This is because the goal of such practices is not a finished product (the fully ‘responsible agent’) but rather an increasingly healthy “moral ecology” of responsibility (2013) in which there is sufficient exercise of responsible agency to underpin social trust and shared flourishing.

What lessons from the instrumentalist account can be applied to the AS responsibility gap? Initially, this looks very promising. For one thing, the forward-looking and relational focus of the agency cultivation approach seems to lend itself to a cautious optimism about our ability to use social feedback to scaffold, guide and motivate the building of better autonomous systems, even if they continue to lack many of the capacities our intuitions tell us that morally responsible agents must have. Such systems need not become *individual bearers* of moral responsibility, since it turns out that arguably even we humans often cannot manage that alone. We need only find a way to cultivate autonomous systems that play a more useful and trustworthy role in a healthy moral ecology of responsibility.

To that end we are justified in creating new and different responsibility practices adapted to AS modes of action; these need not and should not mirror those of individuals.^9^ It can make proleptic sense to direct moral indignation at a human who has caused grave harm; it makes no moral sense to direct that attitude toward an autonomous vehicle or robot that has done the same. Yet that need not close off *other* potential avenues of constructive dialogical engagement and social feedback that are more conducive to building AS that support rather than degrade our shared moral ecology. This view is also in general sympathy with the work of Nyholm (2017), Coeckelbergh (2020); Tigard (2021a), who offer comparable responses to the AS responsibility gap that rely on hybrid, pluralistic, ecumenical and/or relational accounts of moral agency.

Our view differs from Gogoshin’s (2021), who also responds to the AS responsibility gap by adopting the instrumentalist view of moral responsibility. Gogoshin offers a behaviourist model of responsible machine agency, arguing that this can be usefully separated from the role of reactive attitudes and affective responses in instructing and shaping moral behaviour. She argues that in the case of AS a sufficient condition for both responsible agency and membership in the moral community is “the capacity to reliably behave according to moral norms” (2021, 5). She holds that since there are plausible engineering methods for influencing machines to produce morally competent behaviour, this is sufficient to cultivate morally responsible machine agency.

We reject this purely behaviourist approach. Most fundamentally as Jefferson (2019) notes, on the new instrumentalist accounts, responsibility practices do not just aim to improve moral behaviour. They aim to foster the development of moral *agents*. We do not think that today’s AS can *themselves* be moral agents. Even if we were to succeed in using engineering feedback mechanisms such as inverse reinforcement learning (Hadfield-Mennell et al. 2016) to get them to reliably mimic moral behaviour, this only enables this class of artefacts to play a more constructive and stabilising role in the moral ecology of responsible *human* agency. We are not cultivating a *new* class of moral agents. This is because the moral performance of AS tools can only be improved through the understanding of moral reasons by their designers, users and regulators. Their ‘moral performance’ is entirely derivative, as the system as a unit lacks its own access to moral phenomena such as concern, trust, need, care, obligation and vulnerability.

Moreover, the derived moral performance of a machine does not issue from an active moral *skill* of the sort that responsibility practices aim to cultivate in individual agents. If being a responsible agent is a skill that agents actively maintain in light of their understanding of moral norms, which in turn is constantly updated by social feedback, then it is highly unlikely that a system that neither understands norms nor the reasons behind social feedback can develop and maintain such a skill. Systems can be made to have better or worse behavioural tendencies, but they cannot actively maintain skills that require moral judgment and the understanding of social feedback. As Milam (2021) notes, even influence theories of responsibility, which valorise the construction of responsibility by influences external to the agent, require those influences to act upon the person’s *own* moral agency, not upon a custodial or morally insensate mechanism. Influence theories will not hold asteroids to have responsible agency just because they can be steered out of Earth’s path, nor animals to have responsible agency just because they can be restrained by their owner’s command (449).

Human designers and users within AS *are* obviously well capable of understanding moral reasons and using that knowledge when designing or operating the system. Hence we are far more sympathetic to Coeckelbergh (2021) and Tigard (2021a)’s views on the need for AS, viewed from the wider sociotechnical systems lens that includes their human enablers, to become more *answerable* to the patients of responsibility they impact. For while AS are certainly not moral agents, the human parts of these sociotechnical systems very clearly are.

It is the humans within an AS, who can come to more fully understand and appreciate the weight of moral considerations in a way that the machine assemblage cannot, to whom we address our responsibility practices. It is the human (individual and collective) agency behind AS that such practices must improve. As Tigard notes:

We hold others to account by communicating wrongdoing and even punishing, but not necessarily because they deserve to suffer. Very often, we want to see that the future is better than the past. We want to help others learn from their mistakes and discourage repeated harmful behavior. (Tigard, 2021b, p. 603)

This then is the outline of a solution. Once the question of holding agents responsible moves away from a backward-looking attribution of blame, and focusses instead on the forward- looking fostering of more responsible agency in our shared moral ecology^10^, then it seems that we have many more options for bridging the AS responsibility gap. We can and should still hold the appropriate humans responsible for blameworthy AS design and use, but even in cases where we cannot establish backward looking blame, an open-ended suite of other responsibility practices remains available to us, including answerability and accountability practices understood proleptically.

The concept of answerability has been used in the responsibility literature in multiple ways; sometimes to capture a particular dimension of responsibility (Shoemaker, 2011), sometimes as a way of characterizing responsibility as a whole (Smith, 2012). As we have seen, on Shoemaker and Tigard’s accounts, answerability describes the capacity and duty of a responsible agent to offer a rational justification of their conduct. Yet following Duff (2021), we adopt a more expansive view of answerability. For as Kiener (2024, 208) notes, we can be legitimately answerable even in cases where we are incapable of supplying the answer in the demanded form (a rational account). If I strike you without even thinking, I will recognize your right to demand my reasons for doing so even if I have none to give, even if my action seems to me to have had a completely alien, ineffable source.

In some cases I am answerable even for actions that are not mine. For example, if my friend reasonably but wrongly assumes that I caused the fresh damage to her car that I borrowed this morning, the trust relation between us makes me answerable insofar as I am, at a minimum, obliged to explain that I witnessed a passing car sideswipe it after I left it safely parked. While my friend’s indignation toward me is no longer justified once this excuse is given, my role in the chain of events that begun with my friend’s generosity keeps me ‘on the hook’ to answer in other ways, for example, to help her make a detailed report to the police. These more expansive duties of responsibility are elaborated more fully by Duff, who observes the rich diversity of answers that responsible agents can be called to provide in different situations, well beyond rational explanations and justifications. In some cases a harmed party may have no moral interest in my explanation, and may be far more invested in obtaining from me a sincere apology, a meaningful remedy, or an act of reform.

The more expansive notion of responsibility as answerability can be well justified by instrumentalist accounts, insofar as these forms of answerability promote responsible agency cultivation. My duty to answer my friend, even for a harmful act that was not mine, is part of building up my capacity to be a responsible borrower of another’s property, to show my care for what is entrusted to me, and to do the essential work of maintaining relational bonds that support a healthy moral ecology. As Kiener notes, we can think of answerability practices as cultivating a certain virtue of ‘moral ambition’ to responsible agency (2024, 214).

Answerability also enables us to respond more fully to the responsibility gap challenge for AS. For while the obstacles to satisfying the epistemic and control conditions for AS may impede our capacity to explain some system actions—just as they do with many human actions—these are not obstacles to many other kinds of answers that the humans behind the AS can rightly be asked to supply to those harmed by such actions, especially proleptic answers that anticipate more responsible ways of using AS in the future. Humans can be called to answer even for many kinds of AS acts for which they are not blameworthy.

However, our view retains the traditional link between answerable agency and the appropriateness of the reactive attitudes, insofar as the latter must serve a motivational purpose. Not just *anyone* can be called to answer for an AS harm I have suffered, and not just anyone can be a legitimate subject of reactive attitudes arising from that harm. It must be someone whose capacity for responsible agency is implicated in some appropriate way by that harm, even if they are not rightly subject to blame. As we will see in the next section, our responsibility practices presuppose that the agent in question is *vulnerable* to our reactive attitudes in such a way that they can be moved to become more responsible as a result.

This exposes a key asymmetry between humans and AS with respect to answerability practices: the asymmetry of their *vulnerability* to the moral relation. As we have seen, the key feature of practice-first, instrumental accounts of moral responsibility is the dialogical nature of the practices that drive and monitor the skillful acquisition, honing and maintenance of the abilities required for responsible agency. As Strawson, and McGeer following him, point out, this dialogue is driven by our reactive attitudes.

Reactive attitudes such as moral outrage and indignation (expressed by the patient or wider moral community) and moral shame and remorse by the agent, are the motivational fuel that gives responsibility practices their oomph. This is no less so if we see the function of these attitudes as proleptic rather than purely backward-looking. Even if I could not have foreseen or prevented a harmful past action of mine, the moral indignation of my victim can motivate me to take steps now to ensure that it will not happen again.

But this creates a problem in the case of AS. Even when viewed as sociotechnical systems rather than mere machine assemblages, they don’t seem be the right kind of entities to get the responsibility dialogue off the ground. They are neither able to feel moral emotions themselves nor are they receptive to the moral emotions of others. The machine components of an AS are intrinsically incapable of it, and as we explain in Section Four, the individual humans within an AS are often blocked by organisational structures from that receptivity and response of which they are otherwise capable. In other words, sociotechnical systems are typically not *vulnerable* to the powerful effects of negative moral emotions of those they harm, nor can they project moral emotions adequately in response. It is this invulnerability and moral inertness that we see as the more significant responsibility gap with AS.

## The Vulnerability Gap in AS

Tigard’s (2021a) analysis of *technological answerability* acknowledges the central role of dialogue between agents and patients. Following McKenna’s conversational model (2012), Tigard notes that “the process of demanding, giving, and receiving answers can be rightly considered a paradigm of a *moral responsibility exchange*… (9)”. In the case of AS, he proposes enhancing the system’s capacity to give answers that reduce the risk of our technological ‘severance’: that is, explanations, reasons or justifications that enable patients of system actions to better understand “why things happen” (15), bolstering the link between their own agency and the “technological processes at work around them” (11).^11^

But this model of a responsibility dialogue as an *informational* exchange that primarily facilitates our understanding of the links between action and consequences is stripped of a key dimension. It does not adequately address the role of the moral emotions and mutual vulnerability in responsibility exchanges. Responsibility practices for humans are a high stakes enterprise that we cannot escape if we want to be accepted by others as responsible and trustworthy agents. That is, humans are morally responsible to one another because we are physically, emotionally, cognitively and politically vulnerable to one another’s powers, and because our voluntary uses of our power can impinge upon others’ vulnerabilities in ways that cause harm and undermine the social bonds of trust, cooperation and mutual aid upon which human flourishing depends.^12^

In responsibility practices these vulnerabilities manifest themselves not only in the acknowledgment of the potential harm done to the patient of responsibility (i.e., the subject impacted by the action) but also in the recognition of the vulnerability of the *agent* to negative reactions to their own action, arising from the patient and wider moral community. For this reason, patients of responsibility have their own moral duties to the agents they seek to hold accountable; for example, they must treat the agent with some moral respect, listen with care to the agent’s answer, and be ready to respond in turn (Duff 2018).

In fact, a core purpose of responsibility practices is to not only recognise the harm done to the patient, but to negotiate appropriate consequences of the action *for the agent* in the mutual recognition of each other’s vulnerability. Responsibility practices cultivate responsible agency in humans by shaping and constraining our dispositions to use our power. Their effect on me as an individual is not only the protection of vulnerable others from further harm at my hands, but my *own* protection, as the agent, from serious affective and social injury: the shame, guilt, alienation and exclusion that may result from the abuse of my power.

AS do not face comparable vulnerabilities. As noted by Scheutz (2011) and others, the power asymmetry produced by their affective invulnerability to our social practices of interpersonal regulation poses a significant risk to us, and is a serious impediment to building trustworthy systems. It goes without saying that the machine components of an autonomous system, as nonsentient artefacts, cannot directly experience or enter into a mutual recognition of human vulnerability and its morally obliging force. In addition, while AS are also *socio*technical systems, that is, always partially enacted by individual humans and groups who design, develop, deploy and maintain them, the humans in an AS do not typically occupy the right kind of relational roles to instantiate the vulnerability recognition that anchors moral responsibility and interpersonal trust.

This is because most of the time the ‘socio’ part of the sociotechnical system is not enacted by an individual human agent, but by a large number of people with varying and limited degrees of involvement in the creation and use of the machine components. The actions of both the individual humans and the machine assemblage are directed by multiple teams and organisations within an extended supply chain and deployment environment, that interact in diffuse, indirect and complex ways (Cobbe et al., 2023). The consequence of this fact (known as the ‘many hands’ problem) is that typically, no human occupies a role in an AS that would be comparable to an individual agent in a responsibility exchange. No individual human in the collective has the unique moral standing to negotiate appropriate consequences for the whole sociotechnical system in the future, and more importantly no human is tied to the past and future system’s actions, and the responses of patients to them, in the same inescapable and deeply vulnerable way that an individual agent would be.

For example, consider the responsibility relation between a clinician and her patient receiving the clinician’s diagnosis.^13^ Here there is shared knowledge and mutual recognition of the vulnerabilities at stake (the health, comfort and survival of the patient, *but also* the reputation, confidence, financial security, legal exposure and professional status of the clinician). The clinician and the patient can recognise and are answerable to one another in different ways as a result of these vulnerabilities. Compare this with a hypothetical situation in which the patient uploads a set of biometric readings or lab results and receives a diagnosis from an autonomous system. Imagine that the medical professional in this context is not patient-facing but only evaluates the system’s performance over time by periodic sampling and checking of its diagnostic results. Now imagine that in both cases there is a diagnostic error resulting in the patient’s permanent disability. How are these two cases different from the standpoint of a moral responsibility exchange?

We argue that most morally significant difference from the standpoint of moral responsibility is *not* that the medical professional in the second case lacks knowledge or control of the process of generating the patient’s diagnosis. While these do compound the difficulty of appropriately answering for the action, they are common challenges, and they complicate but do not typically impede responsibility ascription. For imagine a plausible scenario in which the clinician in the first case is not sure, even in hindsight, why they missed the correct diagnosis. They might fabricate a guess, but suppose they simply do not know, with any degree of confidence or precision, why they judged the patient’s cluster of symptoms and lab results as indicating a different disease. They just did. Yet there is no question that they *are* the responsible party, and they remain vulnerable to the consequences of their failure.

Questions of blame, and what is owed to the patient, will turn on further facts; but even if it turns out to be a blameless error from a legal and/or moral perspective, both the patient and the doctor know who is the *responsible* party, that is, who must answer for it (even if the answer is exculpatory). Moreover, as long as the negative affective and social reactions to the clinician are morally appropriate, not exaggerated or misdirected, they remain able to shape the clinician’s agency in such a way as to enhance their future responsibility. For example, the patient’s expression of pained betrayal may make the clinician more discerning in future similar judgments (whether or not they are conscious of the mechanism), or may boost their epistemic humility, leading to more frequent cross-checking of guesses with a colleague.

Yet in our second case, the patient will not know who must answer for the erroneous diagnosis, and it’s not clear that the medical evaluator of the AS will know themselves to be the one responsible, i.e., answerable, to the patient. Nor is it clear that they *are* the answerable party, at least not in the same way as the clinician in the first case, who is answerable to the patient even if they cannot understand their error, or how they might have avoided it. For the medical evaluator in the second case will be merely one of a *team* of professionals, including software engineers, data scientists and machine learning researchers, tasked with evaluating the system’s overall performance, all equally disconnected from the injured patient.

Why should the medical evaluator be the one to answer the patient, to be vulnerable to their resentment or pain? Why not the model builder, or data scientist? Will any one of them see themselves as having a clear duty to answer to this one patient, who they’ve never met? *Should* they? Yet someone must have this duty, or else *there is no responsibility exchange*. The salient difference between our two cases is that none of the professionals on the AS product team in the second case seem to stand in the kind of role relation appropriate to an answerable party, namely a party who was expressly or implicitly trusted by a patient to exercise care with their vulnerability, and who must make themselves vulnerable in turn to the patient’s response.

A further variation of the scenario nicely illustrates that the responsibility gap here is a many hands issue, and not the lack of agent control and knowledge as traditionally assumed. Imagine that the medical professional getting the results from the AI in our second case is not only a medic but also the inventor, builder and sole deployer of the system. In this case they might be equally unsure why the system gave the diagnosis it did, but in sharp contrast to our original case, there seems to be no question here that our inventor medic is the answerable party, and rightly vulnerable to and obliged to deal with the justified demands of an injured patient.

As the above makes clear, the vulnerability gap – the absence in an AS of any identifiable agent standing in the right kind of trust relation to a vulnerable party, and themselves vulnerable to the relation—is hardly unique to AS. It’s a more complex example of a known failure mode of many existing institutions and bureaucratic systems, where the agency behind one act is broken up into smaller processes distributed among ‘many hands’ and ‘many things’ (Coeckelbergh, 2020), none with the right kind of answerability relation to the vulnerable others upon whom the system ultimately acts. It is much the same problem that appears in the context of the moral responsibility of corporations, which also stand in asymmetrical vulnerability relations to those impacted by their decisions. Thus solutions to the AS vulnerability gap will likely lie in the same direction as solutions to institutional and organisational distance from human vulnerability.

Yet even if the vulnerability gap is not unprecedented, AI industry-fueled growth in the power and scale of autonomous systems is making a bad situation worse. For it contributes not only to the wider *distribution* of agency, something that multinational corporations already do, but even more to what one might call agency *disintegration*. That is, AI technologies allow the greater fragmentation of formerly coherent acts or processes defined by specific motivations and purposes for which people or organisations can be held accountable. When embedded in AS, they often disperse the contributions of human will, introduce more chaotic and random effects in action chains, and sever the cognitive and motivational links between means and ends that give actions moral meaning.

Consider, for example, the disintegration of agency that results from new generative models for image creation, such as DALL-E or Midjourney. In place of a process where a human artist deliberately selects various means (tools, techniques, style, perspective and media) to attain a desired image, today the human ‘artist’ may simply enter a text prompt, such as ‘a painting of a sad turkey riding a bicycle on a pointillistic moon.’ The AI tool will then create a number of possible variations on that theme, from which the artist can then choose and make further manual edits before submitting that image to a contest, a gallery, or a client.

The human creator is still ‘responsible’ for the image by the traditional ‘knowledge and control’ accounts of responsibility. They know what prompt they used, what image they select, and maintain full control over what is done with the selected image. But there are gaps in the artistic process where genuine aesthetic agency (most notably aesthetic motivation or will) has dropped out, leaving only the mindless power of the tool to make key decisions. The deleterious effects on the health of responsibility exchanges can be seen in online conversations where creators using these tools show affective indifference to social criticism of their work’s parasitic appropriation of other artists without credit. The same artist who would instantly redden with embarrassment and shame at being publicly exposed for stealing from a fellow artist’s catalog has a far harder time feeling these moral emotions when the tool, not they, did the stealing.

This agency disintegration can be compounded by AS at multiple levels in the moral ecology, much in the way that concentrations of a toxin rise in the food chain. For example, an art contest judging panel can now use algorithmic filtering and ranking to reduce a large set of submitted images to a smaller group for human consideration. The human panellists are still nominally responsible for the ultimate choice of the winner, but the mediation of the end-to-end process by AI makes their group agency strangely intermittent or ‘gappy.’

Where this becomes particularly troubling from the standpoint of responsibility is where this disintegration of agency combines with the ‘many hands’ phenomenon to make human responsibility merely nominal and perfunctory, unable to perform its true function in grounding social trust. Take, for example, the airline desk agent who is tasked with assisting stranded passengers or those whose bags have been waylaid. Today, the automation of key airline systems combined with the distribution of actions and policies across a large corporate network results in a phenomenon where the human desk agent who is nominally ‘responsible,’ that is, answerable to a customer, can in fact do virtually *nothing for them.*

Whereas a decade ago a desk agent will have had the power to call an operations number and advocate on behalf of passengers, or present them with choices to consider, often today an agent will have no insight into the passenger’s particular circumstances or options, no one they can call, and little power to do more than point the passenger to an automated app which may or may not tell them where their bags are or how to get to their next destination after a flight cancellation. Even if the human agent is empowered to offer the passenger some limited assistance, however, they remain distanced from the passenger’s particular plight in a way that undermines the relation between them.

This is because while the passenger may have ample reason to be angry about their circumstances (for example if they have been stranded without cause, notice or remedy), they know they cannot fairly direct this anger at the desk agent, who quite literally *has almost nothing to do with it*. The desk agent can look at a screen and confirm what the passenger already knows, but they have neither caused, nor have any further knowledge about, nor are empowered to remove the obstacle to the passenger’s travels. They sit atop a layered network of automated actions in which they are in no way implicated, either in a backward looking or forward looking sense. They are not therefore a legitimate subject of reactive attitudes like blame or resentment, and both they and the passenger know it.^14^ This is related to but distinct from the “retribution gap” between machines and humans noted by Danaher (2016). Here there *is* a human moral agent who can be a target of the passenger’s retribution, but they have no agency that is pertinent to the passenger’s situation. They remain emotionally vulnerable to the passenger’s response, but this vulnerability is no longer constructive of trust or future responsible agency. Notably, this also constitutes a moral injury to the airline representative, who is deprived of the ability to cultivate and express their moral agency in a situation that demands it.^15^

AI systems used to expand automated decision-making will only worsen such breakdowns in our moral ecology, by further disintegrating agential processes that are already distributed among many hands. For example, an AI system will decide which alternate flight to rebook the passenger on, or how to reroute their lost bag; the human desk agent is now only the messenger; no more than an organic display screen. Yet due to the lack of sentience and common-sense reasoning in AS, they rarely automate entire jobs or end-to-end processes. Instead they are more often being used to automate parts of processes, tasks or decisions that sit within a larger role. Every time this happens, whether with a car accident, a cancer diagnosis, or a decision to shoot someone in war, we have created a new instance of the vulnerability gap where there had been none so far. In order to preserve the health of our moral ecology and the social trust and cohesion it sustains, we need to determine how best to negotiate or bridge this gap, or at least avoid widening it.

## Objections and Conclusions

An objector might concede that AS, even as sociotechnical systems, are not vulnerable in the way individuals are,^16^ but argue that this is less problematic for responsibility practices than we make out. Why not use the lessons from the agency cultivation approach to create new forward-looking practices designed to mitigate this moral severance? One attempt to do something like this for the problem of corporate or group responsibility comes from Astola (2022) who argues for a virtue ethical approach to collective responsibility. Astola suggests moving the collective responsibility discourse away from seeking a mechanism to assign backward-looking blame to groups for past actions, and toward identifying mechanisms to help collectives develop stable character traits that would make it less likely in the future that such blameworthy events will happen.

Such mechanisms would aim to foster collective interest in becoming a responsible corporate agent, and the shared moral knowledge and motivation to take steps to make this happen. This doesn’t directly address the AS gap, partly because on Astola’s view, responsible groups must cultivate a *collective identity.* AS, on the other hand, are typically enabled and deployed by many diverse organisations. Moreover, members of a virtuous but heavily automated collective may be no more empowered than our helpless airline agent to respond appropriately to a patient’s moral emotions about a harm. A sincere ‘We are very sorry that happened to you, and we aim to do better’ may be all that they are individually empowered to offer, and in many cases this will not be sufficient. If patients exposed to the power of collectives, and now to AS, are increasingly cut off from the vital communicative practice of reciprocal moral address, the problem of moral severance and its social costs remains.

Where does this leave us? We have established that attributing responsibility to AS in some ways faces surprisingly similar challenges as in the case of human individuals and collectives. This is because individual human agency is both far more socially embedded and increasingly more distributed than traditional accounts of responsibility assumed. While evidence from the cognitive sciences suggests that the knowledge and control gaps typically seen as the roots of the problem for AS are in fact reproduced in mundane human contexts, AS do widen existing responsibility gaps created by increasing corporate and institutional distribution of agency. They also increasingly drive the disintegration of agency, which places even greater pressure on traditional responsibility norms and practices.

The good news is that agency cultivation approaches allow us to reconceive responsibility practices as compatible with widespread individual deficits in knowledge and control. Moreover, the constructive and instrumental nature of responsibility practices on this view entail cautious optimism that we have the resources and flexibility to craft *new* responsibility practices better adapted to the challenges presented by AI and autonomous systems. We therefore endorse the ideas of those who propose developing new ways of enhancing the answerability of AI and autonomous systems to people; certainly by finding better ways to make the human actors embedded in those systems answerable for AI’s power (Nyholm 2017, Coeckelbergh, 2020), but perhaps even by enabling the machine components of the system to provide more limited answers as appropriate to their capabilities (Tigard, 2021a).

But while this is important progress, we argued that even agency cultivation approaches do not provide us with a straightforward solution of the responsibility gap, because they provide no obvious prospects for addressing the growing asymmetry of vulnerability between agents and patients in the vital responsibility practice of moral address. We argue that this kind of reciprocal and affectively-laden moral address is valuable for more than its ability to motivate moral compliance with social norms. Even if that function could be replaced by other means, the communicative practice of moral address between mutually vulnerable persons plays a central role in the formation of human moral identity and self-understanding, the building and sustaining of social trust, and the virtues of care and solidarity. The growing disruption of our moral ecology by machines that cannot engage in this practice, and which increasingly sever us from it, remains a serious cause for worry.

This leaves us with a precise diagnosis of the problem but incomplete indications of the shape of a solution, something we hope to address directly in future work. What any solution to the vulnerability gap must achieve is a path for AI and automated systems to support a stable moral ecology, in which the practice of meaningful and reciprocal moral address is secured.

## Footnotes

1 Here and throughout the paper we follow a definition adopted more widely within the UKRI Trustworthy Autonomous Systems programme, of which our project is a part: https://tas.ac.uk/our-definitions/. This engineering notion of autonomy stands apart from the moral/philosophical one; autonomous and semi-autonomous machines are those that can complete a task or take action more or less independently of human supervision or direction. These need not have any of the elements of rational autonomy that ground, for example, the dignity of persons in Kant.

2 For a recent summary of the debate, see Oimann (2023).

3 Arguably, autonomous systems driven by machine learning algorithms require *more* human agency to operate successfully than older, nonprobabilistic AS such as factory robots; this was anticipated by Bainbridge (1983).

4 While a small minority of researchers might contest this claim, it represents the current mainstream consensus among both computer scientists and moral philosophers.

5 Kiener’s approach mirrors that of Michael Davis’ (2012) account of engineers’ responsibility, where this responsibility-taking can be manifested in a voluntary act of professional virtue.

6 Heinrichs (2022) ends up in a position of skepticism with regard to the aptness of the responsibility concept in this context; for reasons articulated in Section Three, we hold that this ignores the vital and indispensable function of responsibility in the moral ecology, and specifically in shaping human moral agency.

7 This is one reason why, as explained more fully in Sect. 3, we favor the broader notion of answerability developed by Duff (2021), as his account allows for cases where answers are rightly expected from and given by agents even when their reasons and justifications for acting are inaccessible or morally irrelevant to us.

8 It should be noted here that our focus on the proleptic skill-based account of responsibility is probably best aligned with McGeer or similar positions like Jefferson (2019) than positions like Vargas which maintain at least some elements of the classical knowledge and control condition and are therefore not as immune to worries related to those. Thanks to an anonymous reviewer for pressing us on this point.

9 Our reliance here on instrumentalist approaches to responsibility is pragmatic; while theoretical alternatives persist, instrumentalist solutions are particularly suited to fill the lacuna in the need for justification of new responsibility practices for autonomous systems. As discussed earlier, philosophers of AI sometimes argue that we do not need to worry about the epistemic or the control condition in the AI context, because we demand answers of humans despite the fact that humans also often do not fulfil these conditions. But there is not enough attention paid to the question of why we are *justified* in demanding answers of both humans and machines, even when these two conditions are not fulfilled. The instrumentalist approach provides this justification.

10 That this forward-looking approach also chimes with our intuitions is nicely illustrated by the fact that victims of crime and other blameworthy actions very often care deeply about ensuring that their suffering has not been in vain. They might e.g. get engaged in campaigns to change laws or processes so that their suffering is not experienced by others in the future. In some cases the relevant laws even get named after the victims of the initial harm.

11 He rightly notes that patients often want *other* kinds of answers, which it is less clear that machines can provide: acknowledgements of harm, apologies, remedies and promises.

12 See also Vallor and Ganesh (2023).

13 Here is a *patient* in a dual sense: the conventional medical one, and the one that describes anyone vulnerable to another agent’s power of action.

14 The agency diffusion and disempowerment driven by many AI-enabled automated systems also blocks the ‘vicarious responsibility’ via ‘moral entanglement’ solution to responsibility gaps suggested by Goetze (2022), since unlike an apologetic parent or dog-owner, no one human agent may be strongly entangled with a system action, or empowered to shape the system’s future behaviour in a meaningful way.

15 Another kind of disruption to our moral ecology of responsibility practices is noted by Vargas (2021, 439). Social media platforms have greatly reduced the cost of blaming others, which creates vulnerability gaps in the other direction. For example, anonymous or mass brigading, doxxing, and related practices often enable invulnerability on the side of the blamer, who no longer has to stand behind their accusation or respectfully listen to—or even acknowledge—an exculpation or justification by the blamed agent. In turn, individuals and especially corporations are becoming less responsive to online blame; targeted individuals increasingly tend to delete their account rather than try to defend themselves, and gone are the days when one could expect a company’s Twitter account to quickly apologize or offer a meaningful remedy for a harm gone viral.

16 Some might not even want to concede that much. Björnsson and Hess (2017) argue that corporations can feel moral emotions and therefore be full partners in our moral ecology. There is not enough space to argue with this in detail but we do not think that their account works, because the moral emotions and vulnerabilities of the individuals in the companies do not relate to the aims of the company in the same way as they would in an individual. But even if Hess & Björnson’s radical proposal could be made to work it would still fall short of solving the AS vulnerability gap, because AS are often not operated by one structured corporation but are spread out among a very diverse set of institutional and individual agents.

## References

[CR2] Andersson, O., et al. (2017). Subliminal influence on generosity. *Experimental Economics*, *20*, 531–555. 10.1007/s10683-016-9498-8

[CR3] Astola, M. (2022). Collective responsibility should be treated as a virtue. *Royal Institute of Philosophy Supplements*, *92*, 27–44. 10.1017/S1358246122000133

[CR4] Bainbridge, L. (1983). Ironies of automation. *Automatica*, *19*(6), 775–779. 10.1016/0005-1098(83)90046-8

[CR5] Björnsson, G., & Hess, K. (2017). Corporate crocodile tears? On the reactive attitudes of corporate agents. *Philosophy and Phenomenological Research*, *94*(2), 273–298. 10.1111/phpr.12260

[CR6] Bortolotti, L. (2018). Stranger than fiction: Costs and benefits of everyday confabulation. *Rev Phil Psych*, *9*, 227–249. 10.1007/s13164-017-0367-y. 29904434 10.1007/s13164-017-0367-y PMC5986841

[CR7] Cobbe, J., Veale, M., & Singh, J. (2023). Understanding accountability in algorithmic supply chains. In *Proceedings of the 2023 ACM Conference on Fairness, Accountability, and Transparency* (FAccT ‘23). Association for Computing Machinery, New York, NY, USA, 1186–1197. 10.1145/3593013.3594073.

[CR8] Coeckelbergh, M. (2020). Artificial intelligence, responsibility attribution, and a relational justification of explainability. *Science and Engineering Ethics*, *26*, 2051–2068. 31650511 10.1007/s11948-019-00146-8 PMC7417397

[CR9] Danaher, J. (2016). Robots, law and the retribution gap. *Ethics and Information Technology*, *18*, 299–309. 10.1007/s10676-016-9403-3

[CR10] Davis, M. (2012). Ain’t no one here but us social forces: Constructing the professional responsibility of engineers. *Science and Engineering Ethics*, *18*(1), 13–34. 20686869 10.1007/s11948-010-9225-3

[CR11] Doris, J. M. (2015). *Talking to Our Selves: Reflection, Ignorance, and Agency*. Oxford University Press.

[CR12] Duff, R. A. (2021). Responsibility and reciprocity. *Ethical Theory and Moral Practice*, *21*, 775–787. 10.1007/s10677-018-9898-2

[CR13] Goetze, T. S. (2022). Mind the gap: autonomous systems, the responsibility gap, and moral entanglement. In *Proceedings of the 2022 ACM Conference on Fairness, Accountability, and Transparency* (FAccT ‘22). Association for Computing Machinery, New York, NY, USA, 390–400. 10.1145/3531146.3533106.

[CR14] Gogoshin, D. L. (2021). Robot responsibility and moral community. *Frontiers of Robotics and AI*, *8*. 10.3389/frobt.2021.768092. 10.3389/frobt.2021.768092 PMC8646153 34881294

[CR15] Hadfield-Menell, D., Dragan, A., Abbeel, P., & Russell, S. (2016). Cooperative inverse reinforcement learning. *30th Conference on Neural Information Processing Systems (NIPS 2016)*, Barcelona, Spain. 10.48550/arXiv.1606.03137.

[CR16] Hassin, R. R. (2007). Subliminal exposure to national flags affects political thought and behavior. *Proc. Natl. Acad. Sci. Proc. Natl. Acad. Sci. U.S.A* 104, 19757–19761. 10.1073/pnas.0704679104 PMC2148371 18056813

[CR18] Heinrichs, J. H. (2022). Responsibility assignment won’t solve the moral issues of artificial intelligence. *AI Ethics*, *2*, 727–736. 10.1007/s43681-022-00133-z

[CR17] Hellström, T. (2013). On the moral responsibility of military robots. *Ethics and Information Technology*, *15*(2), 99–107. 10.1007/s10676-012-9301-2

[CR19] Himmelreich, J. (2019). Responsibility for killer robots. *Ethical Theory and Moral Practice*, *22:3*, 731–747. 10.1007/s10677-019-10007-9

[CR21] Hurley, S. L. (2000). Is responsibility essentially impossible? *Philosophical Studies*, *99*(2), 229–268. 10.1023/A:1018763930668

[CR20] Johnson, D. G. (2011). Software agents, anticipatory ethics, and accountability. In: Marchant, G., Allenby, B., Herkert, J. (Eds.), *The Growing Gap Between Emerging Technologies and Legal-Ethical Oversight* The International Library of Ethics, Law and Technology, vol 7. Springer, Dordrecht. 61–76.

[CR22] Kiener, M. (2022). Can we bridge AI’s responsibility gap at will? *Ethical Theory and Moral Practice*, *25*, 575–593. 10.1007/s10677-022-10313-9

[CR23] Kiener, M. (2024). Varieties of answerability. In M. Kiener (Ed.), *The Routledge Handbook of Philosophy of Responsibility* (pp. 204–216). Routledge.

[CR24] King, M., & Carruthers, P. (2012). Moral responsibility and consciousness. *Journal of Moral Philosophy*, *9*(2), 200–228. 10.1163/174552412X625682

[CR25] Köhler, S., Roughley, N., & Sauer, H. (2017). Technologically blurred accountability. In C. Ulbert, P. Finkenbusch, E. Sondermann, & T. Diebel (Eds.), *Moral Agency and the Politics of Responsibility* (pp. 51–68). Routledge.

[CR27] Lim, J., et al. (2017). Moral judgment modulation by disgust priming via altered fronto-temporal functional connectivity. *Scientific Reports*, *7*, 1–14. 28883626 10.1038/s41598-017-11147-7 PMC5589926

[CR28] Luo, Y., et al. (2016). The power of subliminal and supraliminal eye contact on social decision making: An individual difference perspective. *Consciousness & Cognition*, *40*, 131–140. 26821242 10.1016/j.concog.2016.01.001

[CR29] Matthias, A. (2004). The responsibility gap: Ascribing responsibility for the actions of learning automata. *Ethics and Information Technology*, *6*, 175–183. 10.1007/s10676-004-3422-1

[CR30] McGeer, V. (2019). Scaffolding agency: A proleptic account of the reactive attitudes. *European Journal of Philosophy*, *27*(2), 301–323. 10.1111/ejop.12408

[CR31] McGeer, V., & Pettit, P. (2015). The hard problem of responsibility. *Oxford Studies in Agency and Responsibility*, *3*(1), 160–188. 10.1093/acprof:oso/9780198744832.003.0009

[CR32] Milam, P. E. (2021). Get smart: Outcomes, influence and responsibility. *The Monist*, *104*, 443–457. 10.1093/monist/onab011

[CR33] Mudrik, L., Arie, I. G., Amir, Y., Shir, Y., Hieronymi, P., Maoz, U., O’Connor, T., Schurger, A., Vargas, M., Vierkant, T., Sinnott-Armstrong, W., & Roskies, A. (2022). Free will without consciousness? *Trends in Cognitive Science*, *Jul 26*(7), 555–566. 10.1016/j.tics.2022.03.005 35428589

[CR34] Nyholm, S. (2018). Attributing agency to automated systems: Reflections on human-robot collaborations and responsibility-loci. *Science and Engineering Ethics*, *24*(4), 1201–1219. 28721641 10.1007/s11948-017-9943-x PMC6097047

[CR38] Oimann, A. K. (2023). The responsibility gap and LAWS: A critical mapping of the debate. *Philosophy and Technology*, *36*, 3. 10.1007/s13347-022-00602-7

[CR39] Rahwan, I. (2018). Society-in-the-loop: Programming the algorithmic social contract. *Ethics and Information Technology*, *20*, 5–14. 10.1007/s10676-017-9430-8

[CR40] Scheutz, M. (2011). The inherent dangers of unidirectional emotional bonds between humans and social robots. In P. Lin, K. Abney, & G. Bekey (Eds.), *Robot Ethics: The ethical and Social Implications of Robotics* (pp. 205–221). MIT Press.

[CR35] Shoemaker, D. (2011). Attributability, answerability, and accountability: Toward a wider theory of moral responsibility. *Ethics*, *121*(3), 602–632. 10.1086/659003

[CR36] Smith, A. (2012). Attributability, answerability, and accountability: In defense of a unified account. *Ethics*, *122*(3), 575–589. 10.1086/664752

[CR41] Strawson, P. (1962). Freedom and resentment. *Proceedings of the British Academy*, *48*, 187–211.

[CR37] Strawson, G. (2010). *Freedom and Belief*. Oxford University Press.

[CR43] Tigard, D. W. (2021a). Technological answerability and the severance problem: Staying connected by demanding answers. *Science and Engineering Ethics*, *27*(59), 1–20. 34427804 10.1007/s11948-021-00334-5 PMC8383242

[CR44] Tigard, D. W. (2021b). There is no techno-responsibility gap. *Philosophy and Technology*, *34*(3), 589–607. 10.1007/s13347-020-00414-7

[CR45] Tversky, A., & Kahneman, D. (1974). Judgment under uncertainty: Heuristics and biases. *Science*, *185*(4157), 1124–1131. 17835457 10.1126/science.185.4157.1124

[CR53] Vallor, S., & Ganesh, B. (2023). Artificial intelligence and the imperative of responsibility: Reconceiving AI governance as social care. In M. Kiener (Ed.), *The Routledge Handbook of Philosophy of Responsibility* (pp. 395–406). Routledge. 10.4324/9781003282242-43.

[CR46] Vargas, M. (2013). *Building Better Beings: A Theory of Moral Responsibility*. Oxford University Press.

[CR48] Vargas, M. (2017). Implicit bias, responsibility, and moral ecology. *Oxford Studies in Agency and Responsibility*, *4*, 219–247.

[CR49] Vargas, M. (2021). Constitutive instrumentalism and the fragility of responsibility. *The Monist*, *104*(4), 427–442. 10.1093/monist/onab010

[CR52] Waggoner, M., Doris, J. M., & Vargas, M. (2022). Situationism, moral improvement, and moral responsibility. In M. Vargas, & J. M. Doris (Eds.), *The Oxford Handbook of Moral Psychology* (pp. 629–660). Oxford University Press.

